# Migratory timing, rate, routes and wintering areas of White-crested Elaenia (*Elaenia albiceps chilensis*), a key seed disperser for Patagonian forest regeneration

**DOI:** 10.1371/journal.pone.0170188

**Published:** 2017-02-09

**Authors:** Susana Patricia Bravo, Victor Rodolfo Cueto, Cristian Andrés Gorosito

**Affiliations:** CIEMEP. Centro de Investigación Esquel de Montaña y Estepa Patagónica, CONICET/UNPSJB, Esquel, Chubut, Argentina; University of Sydney, AUSTRALIA

## Abstract

Migratory animals often play key ecological roles within the communities they visit throughout their annual journeys. As a consequence of the links between biomes mediated by migrants, changes in one biome could affect remote areas in unpredictable ways. Migratory routes and timing of most Neotropical austral migrants, which breed at south temperate latitudes of South America and overwinter closer to or within tropical latitudes of South America, have yet to be described in detail. As a result, our understanding about how these birds provide links between South American biomes is almost non-existent. White-crested Elaenia (*Elaenia albiceps chilensis)* is a long-distance austral migrant that breeds in the Patagonian Forest biome and overwinters in tropical South America. Because this small flycatcher plays a key role in the regeneration of this ecosystem, our objective was to describe the annual cycle of White-crested elaenias to evaluate the degree of migratory connectivity between breeding and wintering areas and therefore to determine if there are specific biomes of northern South America linked by elaenias to Patagonian forests. Fifteen individuals were successfully tracked throughout a complete migration cycle using miniature light-level geolocators. All individuals resided and moved through the same general regions. During fall (March-April-May), elaenias were located in the Caatinga and the Atlantic Forest biomes, from Rio de Janeiro to the region near Salvador da Bahia, Brazil. During winter (June-July-Aug.), birds were located further inland, within the Cerrado biome. Birds used three different routes during fall migration. Our results indicate that some individuals use a direct route, flying between 500–600 km/day, crossing desert and grasslands, while others took a detour, flying 100–200 km/day through forested areas with refueling opportunities. All birds used the Yunga forest during spring migration, with ten out of 15 individuals showing a clear counterclockwise loop trajectories throughout their annual cycle. None of the elaenias passed through Amazonia, traveled to western South America or crossed the Equator. Eleanias exhibited a high migratory connectivity between breeding area in Patagonian Forests and winter areas, Atlantic Forest and Cerrado. Our results suggest that Patagonian Forests could be strongly impacted by changes in those biomes or in the Yungas.

## Introduction

Historically, research on animal migration has focused on how, when, where, and why animals migrate [1]. More recently, migration studies have also focused on the role of migrants in the communities visited. Given the potential of migrants to link distantly separated communities, ecological processes in one location cannot be viewed in isolation [2]. Migrants function as "mobile links" in community networks, and are essential components for ecological resilience [3]. The strength of interactions produced by migrants can be predicted based on the frequency and duration of their presence at a given site, the resources they consume, and abundance and diversity of other species with similar functional roles [2]. As a consequence of the links between biomas mediated by migrants, changes in one biome could affect remote areas in unpredictable ways [2, 3].

More than 230 species of birds migrate entirely within South America [4], representing the third-largest bird migration system in the world, in terms of number of species [5, 6]. Nevertheless, bird migration in South America is poorly understood [7, 8], such that knowledge about links they provide among South America biomes is almost non-existent. For example, the migratory routes and timing of most Neotropical austral migrants, which breed at south temperate latitudes of South America and overwinter closer to or within tropical latitudes of South America have yet to be described in detail [9]. Most of these species are primarily short distance migrants [4, 5, 10], and as a consequence primarily link adjacent biomes. However, some exceptions exist. For example, Fork-tailed flycatchers (*Tyrannus s*. *savana*) that breed in Argentina accomplish a round-trip migration of 8000 km, connecting "pampas" grasslands in Argentina with "llanos" grassland in Venezuela and Colombia [11].

White-crested Elaenia (*Elaenia albiceps chilensis*; hereafter, “elaenias”) is the longest migrator of Neotropical austral migrant system [12]. It overwinters >6,000 km to the north of its Patagonian breeding grounds, within tropical latitudes of South America [12]. During the Patagonian summer, elaenias breed along a narrow stretch of forest along the Andes Mountains, between northern Patagonia and South America's southern tip [13]. Patagonian forests are ecosystems of low redundancy, biological interactions depend on very few species [14]. Within this biome, 60% of plant species produce fleshy fruits [14], but the frugivore assemblage is relatively poor [15, 16]. During summer, elaenias are the most abundant of the frugivore species [17–19], representing more than 88% of birds captured in the forest during fruiting periods [20], with densities that can reach higher than 8 ind/ha [18]. So, in Patagonian Forests elaenias serves as the main seed disperser [21], and plays a key role in forest regeneration [20, 22].

What are the biomes that elaenias link during their annual cycle? Could impacts to the ecosystems that elaenias use throughout the year negatively impact elaenia and therefore their role in Patagonian forests? There is still no information available about population connectivity of elaenias, and little knowledge exists on the location of their wintering areas and migration routes. What information exists is mainly based on the opinions of experienced ornithologists, field observations, and museum collections. A wintering area in Amazonia has been proposed [23], and Olrog [24] suggested three migration routes for elaenias: one route along the Pacific coast to Peru, a second route that crosses Argentina to the border with Brazil, Bolivia and Paraguay (although with an undefined trajectory), and a third route along the Atlantic coast to southern Brazil. More recently, a migratory route has been proposed along the Brazilian coast, with a wintering area in the Amazon Basin, and with a likely spring migration route south through central Brazil and the Paraguay River [25]. These authors also proposed a route through northern Argentina, with a non-defined trajectory and a migratory route along the Pacific coast to Peru. Some of these routes could represent wintering areas that are used sequentially throughout winter, as was described for other migrants studied with light-level geolocators [11, 26–30]. It is also possible that populations breeding at different latitudes have different wintering areas. More recently, it was proposed that elaenias that breed in the Andean forests of Patagonia overwinter in southern Brazil, migrating through the Espinal biome and along large rivers such as the Paraná and Uruguay [13]. However, La Sorte et al. [31] described the general patterns of migration in the New World based on eBird data (a citizen-science database organized into lists of observed species with the date and location of each records), only described a Pacific coast route for the White-crested Elaenia. If elaenias in fact use a wide variety of routes and wintering areas, then the functional role of elaenias in any particular biome of northern South America should be low, and Patagonian forest dynamics would therefore not be strongly impacted by changes in any particular biome used by elaenias at other times of year. However, only with research on the entire annual cycle of elaenias will we be able to make meaningful advances on the links that they produce among South American biomes. Such research also provides a better understanding of migratory patterns of South American birds in general [3, 8].

Our objective was to describe the annual cycle of the White-crested Elaenia, determining migration routes, rate and dates of migration, and wintering areas, to evaluate the degree of migratory connectivity between breeding and wintering areas. Under a scenario of weak migratory connectivity, we expect that individuals from a specific breeding population use different migratory routes and different wintering areas. Under a strong migratory connectivity scenario, we expect that all individuals from a specific breeding population use similar routes and the same wintering areas. We also aimed to determine whether there are specific biomes in northern South America linked by elaenias to Patagonian forests.

## Methods

### Field methods

We conducted our study at the "Cañadón Florido" cattle ranch (42° 55' S, 71° 21' W) near Esquel, Province of Chubut, Argentina, with permission of ranch's owner. Landscape at Cañadón Florido is mainly woodland dominated by trees of *Maytenus boaria*, *Schinus patagonicus* and *Nothogafus antarctica*.

This work complied with the requirements of the ‘Guidelines to the Use of Wild Birds in Research’ [32]. Ethical and technical evaluation was done by Dirección de Fauna y Flora Silvestre, Ministerio de Desarrollo Territorial y Sectores Productivos de la Provincia del Chubut, Argentina. Field work was conducted during the breeding seasons (October to March) of 2013–2014, 2014–2015 and 2015–2016. We captured elaenias in a 20 ha plot, using three methods. First, we captured birds passively using ten nets (12 m long, 38 mm mesh) placed 70–100 m apart. Nets were opened one or two days every 15 days, between October and March. Nets were opened during the first four to five hours after sunrise when weather conditions were not adverse (i.e. not rainy, windy or extremely cold or hot). Second, we search for elaenias exhibiting territorial displays, and captured them by placing a conspecific model coupled with vocalizations delivered through a portable speaker within 1 m of the mist net (6 m long, 38-mm-mesh). Third, we searched for active nests and captured the parents by placing a mist net (3 x 6 m, 38 mm mesh) and a conspecific model coupled with vocalizations delivered through a portable speaker within 2–4 m of the nest. We avoided capturing the parents during the incubation stage, to avoid abandonment of the nest.

Captured birds were banded with numbered metal bands and a unique combination of up to three Darvic color bands. For each captured individual, we measured wing chord (from the carpal joint to tip of the longest primary), and body mass. All individuals were sexed as described in Cueto et al. [33].

Thirty-five adult individuals were fitted with an Intigeo-P55B1-7 light-level geolocator (0.6 g; [34]) during the 2013–2014 breeding season (14 females and 21 males), and 10 were fitted during the 2014–2015 breeding season (1 female and 9 males). Geolocators were attached using a leg-loop backpack harness [35] made of Filament Kevlar (500 Tex., Saunders Thread, Gastonia, North Carolina). The combined mass of geolocator and harness was <4% body mass of the birds, all of which flew well upon release. All recaptured individuals showed no sign of injury from the geolocator or harness. During the study, the return rate of elaenias without geolocators was 50% and 14.9% for males and females, respectively. Among bird with geolocators, our return rates for males were similar (63%), and higher for females (25%), but we were able to recapture only 14 males of 19 returned and only one female of four returned.

### Analytical methods

Geolocators were programmed to measure light intensity every minute, but to permanently store only the maximum reading every 5 min. Times of sunrise and sunset, calibration, sun elevation angle and positioning were calculated using IntiProc V1.01 [36], based on the GeoLight 1.02 R package [37]. Geolocator calibration and threshold level were estimated by analyzing the light data from the first 5 days after deployment [38], a period during which elaenias were still present at the study site, typically with nestlings or fledglings. This calibration resulted in sun elevation angles ranging from –6.3 to –4.2, and a mean (± SE) error between the location of the birds (hereafter “hotspots", see below) and the location of the breeding site as 58.2 ± 10.5 km.

We estimated two positions per day, and both midnight and noon locations were used in our analyses. The data used in this study are available on Movebank (movebank.org, study name "Migration of white-crested Elaenia (data from Bravo et al. 2017)") and are published in the Movebank Data Repository with DOI 10.5441/001/1.406327g0. We distinguished between movements and stationary periods by inspecting subsequent positions. We defined movements when longitude or latitude showed consecutive, directional changes, and we defined a stationary period when there were at least 14 consecutive positions (i.e., 7 days) showing small changes with no directionality. Following Delmore et al. [39], we rejected obvious outliers and latitudinal estimates near the equinoxes. This equinox period was from 20 days before and 20 days after the autumn equinox for geolocators deployed in 2013–2014, and 10 days before and 10 days after the autumn equinox for geolocators deployed in 2014–2015. For the spring equinox, the period was from 20 days before and 27 days after the equinox for geolocators deployed in 2013–2014, and 10 days before the equinox for geolocators deployed in 2014–2015. We did not set a period after the spring equinox for geolocators deployed in 2014–2015 because they stopped recording ten days after that date.

We created a spatially explicit description of stationary periods of each individual using the heatmaps tools in Qgis 2.4 Chugiak [40]. We set the search radius at 200 km and grid cell size at 2 km. We present a summary map with hotspots (centers of areas including 40% of data,) and heatmaps of each individual location, encompassing 40%, 40–70% and 70–90% of total density. We used Qgis 2.4 Chugiak [40] to estimate migration distances, defined as the straight-line distance between hotspots. Thus, the migration distances we report are minimum distances, and the migration rates represent minimum rates because we defined spring or fall rates as the distance of migration divided by the duration of migration during each period. We also estimated partial rates during fall migration, because there existed substantial heterogeneity in the daily distances recorded.

We evaluated the routes of each individual using longitude information because these estimates are not affected by the equinoxes [38]. As some birds migrated all or partially before or after the equinoxes, mapping the points recorded (latitude and longitude) before and after equinoxes allowed us to define the different routes detected by longitude changes and the biomes used during migration. We used a map of biomes developed by The Nature Conservancy [41] to determine biomes used by birds during stationary periods and *en route*, and used Qgis 2.4 Chugiak [40] to describe the biomes. In figures we are showing schematic maps drawn by us for illustrative purpose only.

## Results

**Fig 1:**
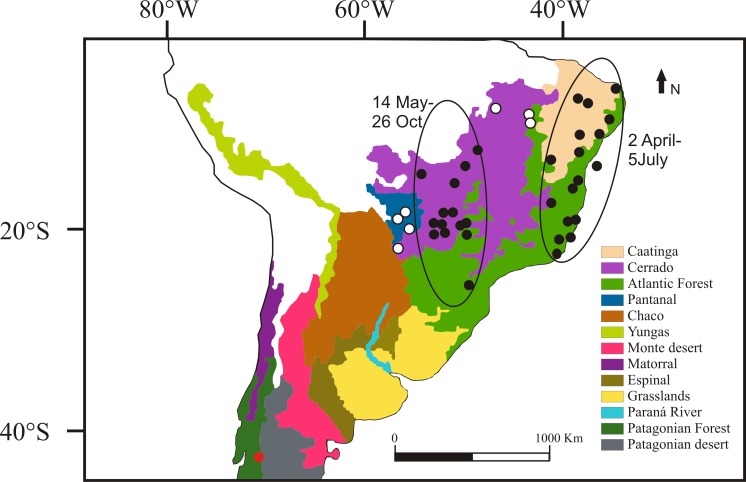
Wintering areas of White-crested Elaenia (*Elaenia albiceps chilensis*). Map of biomes relevant to the elaenia’s annual cycle. Breeding site (red circle), winter and fall areas used by each bird (black circles) are locations with the highest frequency of records by each geolocator, sometimes more than 1 per individual (more details in methods). White circles represent hotspots of areas used by some individuals during more than a week in fall and spring migration.

We recaptured 15 individuals with geolocators (i.e., 33% of the loggers deployed), only one of them was female (H753). Changes in longitude and latitude throughout the year showed that all individuals resided and moved through the same general regions (S1 Fig). During fall (March-April-May), the location of the hotspots indicate that they were in the Caatinga and the Atlantic Forest biomes (Fig 1), north of Rio de Janeiro to the region near Salvador da Bahia, Brazil. During winter (June-July- Aug.), birds were in inland Brazil in the Cerrado biome (Fig 1), although one bird spent time in a southern location in the Atlantic Forest (Fig 1, S2 Fig). No birds crossed the Equator or traveled to western South America (i.e., Peru, Ecuador or Colombia).

### Fall migration

**Fig 2:**
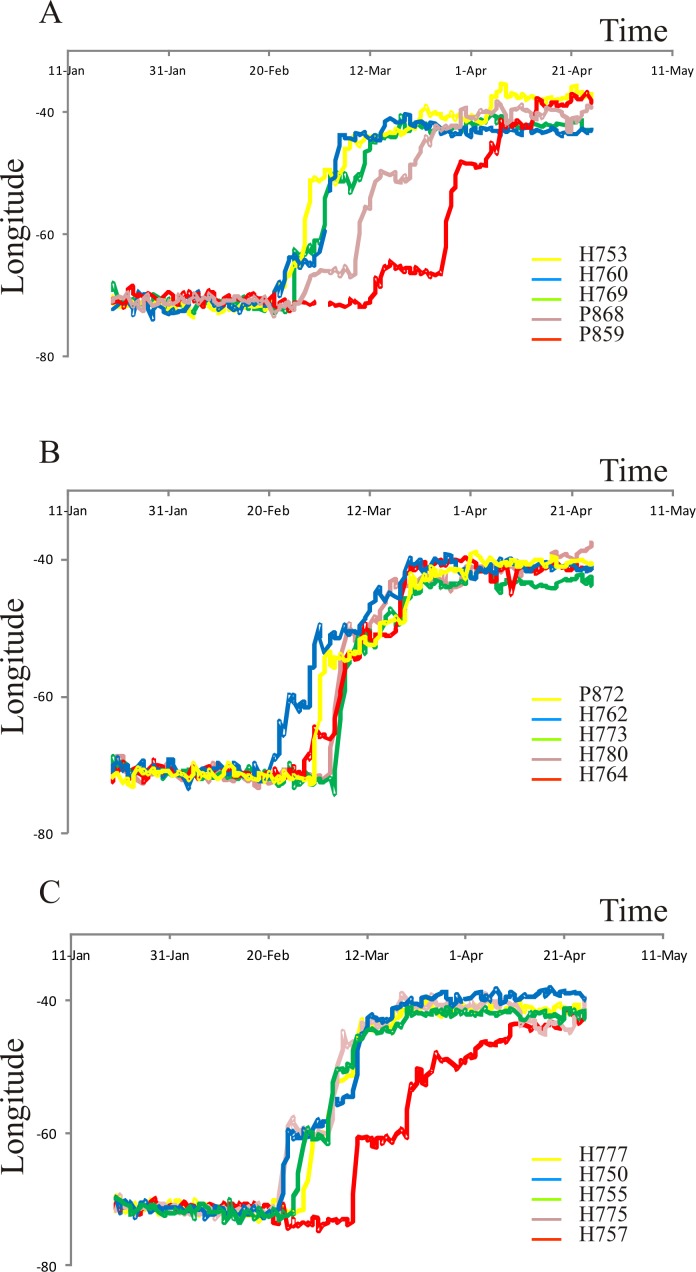
Longitudinal changes during fall migration. We detect three patterns of longitudinal changes during migration. (A) longitude constant during 6–8 days, between 62°W—65°W. (B) changes throughout the entire migration. (C) longitude constant during 6–10 days, between 55°W—60°W.

**Fig 3:**
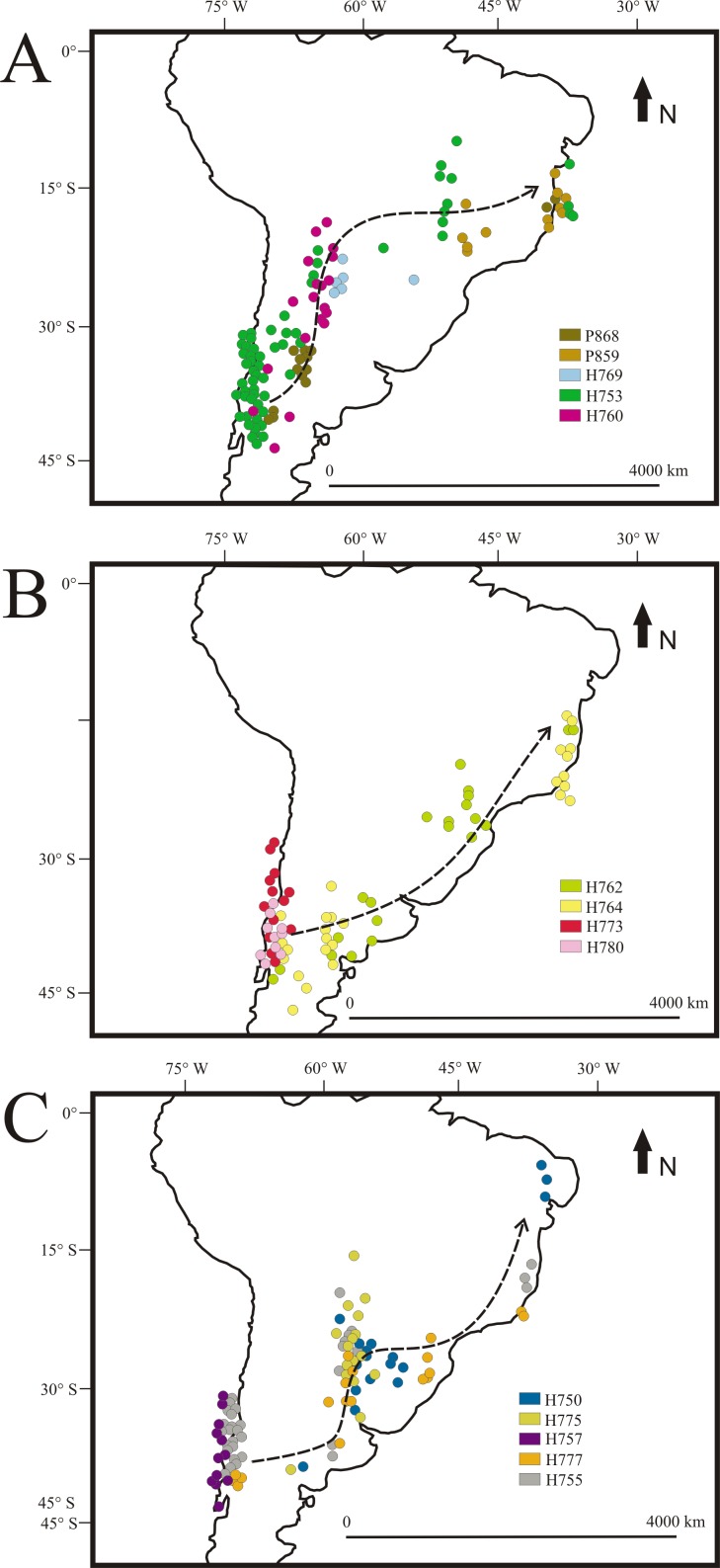
Fall migration routes. Fall migration routes of White-crested Elaenia (*Elaenia albiceps chilensis*). Locations recorded by geolocators during fall migration. The route of individual P872 is not shown because its entire migration occurred during the equinox period. For the same reason, we only show partial data for some birds, for example, for H757, only while it was in the Patagonian Forest. (A) Yungas Route, (B) Coastal Route and (C) Paraná-Paraguay River Route. Arrows indicate the direction and approximate trajectory of migrating birds.

More than 70% of tracked birds started fall migration towards the north using the Patagonian Forest during the second half of February (S1 Table). Birds left the Patagonian Forest between 40° S—32° S latitude, most of them (i.e., 57%) between 22 and 28 February. Longitudinal changes during fall migration showed that birds used 3 different routes when they left the Patagonian Forest (Fig 2). Five birds used a route following the Yungas forest to the north, when longitude was constant during 6–8 days, between 62°W—65°W (Fig 2A). They then crossed Paraguay and southern Brazil, arriving at the Atlantic coast near Rio de Janeiro (hereafter, the “Yungas Route”, Fig 3A). Longitude records decreased during the entire migration in another five birds (Fig 2B), because birds followed a diagonal path, SW-NE. They left the Patagonian Forest, traveling east across the Patagonian desert and the Espinal biomes, then flew north, nearer to the South American coast (hereafter, the “Coastal Route”, Fig 3B). In the third pattern, longitude was constant during 6–10 days at 55°W—60°W (Fig 2C) for five birds. After crossing the Espinal, they followed the Paraná and Paraguay Rivers northwards, until reaching Mato Grosso do Sul in Brazil or Misiones Province in Argentina. They then turned east, crossing southern Brazil to Rio de Janeiro (hereafter, the “Paraná-Paraguay River Route”, Fig 3C). The Coastal Route was the shortest (4,545 ± 562 km), while the Yungas Route was 1000 km longer, and the Paraná-Paraguay River Route was 500 km longer than that.

The average migration rate between the departure and destination sites ranged between 175.0 and 246.5 km/day. However, when birds started fall migration, traveling northwards across the Patagonian Forest, their rate was 31.7 ± 6.3 km/day. They then showed a notable increment in rate after leaving the Andes. We were able to estimate the rate of two birds (H753, H760) along the Yungas Route. Birds traveled ca. 550 km on the first day after leaving the Andes, and while crossing the Yungas their rate was 238 and 182 km/day, respectively. During diagonal trajectories of the Coastal Route and the Paraná-Paraguay River Route, their rate was not constant. Birds traveled 500–600 km/day during one of two days, and on other days traveled only about 100 km/day. Birds traveled greater distances per day while crossing the Patagonian desert and grassland areas. Along the Paraná River, we were able to estimate the rate for two birds (H775, H750), which was 142 and 137 km/day, respectively.

### Winter movements

Between March 11 and April 25, elaenias arrived to the area where they spent the fall; however, although more than 70% of tracked birds arrived during the first half of April (S1 Table). Between May 5 and July 5, elaenias left the coast and traveled to locations within the Cerrado biome to overwinter, although more than 40% of birds left the coast during the second half of June (S1 Table). During this movement, elaenias moved between 1400 and 1500 km during three days, and their rate of movement ranged between 467 and 513 km/day. Three elaenias (H753, H769, H780) departed from the coastal area and used an intermediate area during 7–20 days (Fig 1, S1 Table), migrating between 700 and 1680 km (from the coast to an intermediate location and finally to the Cerrado). This took between 1.5 and 2.5 days, with their rate ranging between 470 and 620 km/day.

### Spring migration

**Fig 4:**
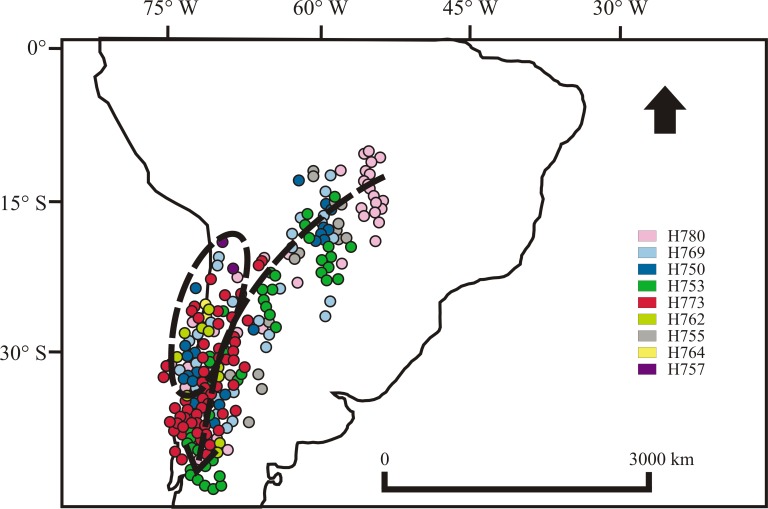
Spring migration route of White-crested Elaenia (*Elaenia albiceps chilensis*). Locations recorded by geolocators during spring migration. We only show partial data for some birds because migration occurred partially during the equinox period. Locations of H777 and H775 are not visible because of the the high density of points. Geolocators P872, P859 and P868 stopped recording before spring migration.

Birds left the Cerrado region between August 21 and October 26, with five individuals spending between 15 and 45 days in the Pantanal (H755, H764, H769, H775, H773, Fig 1, S1 Table). During the second half of October, elaenias arrived in the Patagonian Forest by using the Yungas Route (Fig 4). Before arriving in the Patagonian Forest, some birds spent time along the Pacific coast, traveling to northern Chile (Chilean Matorral) and then returning, but not spending significant time in a fixed location (Fig 4). Spring migration lasted between 13 and 28 days and the average rate ranged between 121 and 261 km/day, and was more or less constant. Considering the entire annual cycle, birds traveled approximately 9800 km. Ten individuals exhibited a counterclockwise loop migration, while five followed the same trajectories during fall and spring migration.

## Discussion

White-crested Elaenias use three fall migration routes and two wintering areas in eastern and central Brazil. All elaenias studied returned to the breeding area in Argentina along the same route. Furthermore, most individuals showed clear counterclockwise loop trajectories. Atmospheric conditions [42, 43] and seasonal changes in ecological productivity [44] drive looped migration strategies, and both mechanisms could influence the migratory trajectory of White-crested Elaenias. This is the first observation of loop migration in a Neotropical austral migrant. However, as we recuperate only one geolocator deployed in females, our results are essentially about male elaenias.

The three routes used by birds during fall migration were in part concordant with routes described previously [13, 24, 25]. However, we did not detect a migratory route along the Pacific coast from our study site to northwestern South America (e.g., Peru). We detected a well-defined migratory route along the Yungas in northern Argentina, while previous studies indicated this region as a diffuse passage site of migratory birds heading to central and northern Argentina [24, 25]. During spring migration, all birds use the same route, traveling through the Yungas.

Some migratory routes suggested to exist based on museum collection data [25] were actually wintering areas where elaenias spent around three months. No birds used Amazonia and contrary to what was proposed by Capllonch et al. [13], our data indicate that White-crested elaenias use the Cerrado and Pantanal biomes. The use of successive winter areas has been reported for other migratory bird species studied with light-level geolocators, and was suggested to be driven by climate or resource availability [11, 26–30]. Elaenias arrived in both the Atlantic Forest and Cerrado during the dry season [45], characterized by a low supply of insects [46, 47]; nevertheless the most abundant plant species of the forest understory (e.g., *Psychotria* spp., *Rudgea jazminoids*, *Rudgea viburnoides*, *Schefflera morototoni*, *Miconia* spp.) have ripe fruits [48, 46]. Shrubs and small trees are generally related to regeneration phases of the ecosystem and produce a high quantity of nutritious fruits that are dispersed by birds [49, 50, 51]. As fruits are important component of elaenias diet [16, 20], the use of successive winter areas seems to be driven by both climate and resource availability.

While undertaking winter movements, some elaenias remained sedentary for several days at a time (7–20 days), and before initiating spring migration some individuals were sedentary for a long time (between 15 and 45 days) in the Pantanal. Additionally, some individuals spent time along the Pacific coast of northern Chile during spring migration. These stopover patterns could be associated with the need to recuperate and refuel during migration (e.g., replenish fat stores). For example, Pyle et al. [52] reported high fat loads of White-crested Elaenias during October in Fray Jorge National Park, north-central Chile. It is very interesting that not all individuals exhibited these stopover patterns, suggesting a need for research on individual variation in elaenia ecophysiology. After those stops in northern Chile, birds moved quickly to their breeding area in the Patagonian forest. This pattern is concordant with the fast movement of elaenias across the Monte desert [53].

The migration rate of White-crested Elaenia was high, in the range of rates reported for the fastest migrants studied [52], for example Purple Martins (*Progne subis*; >150 km/day [54]), European Hoopoes (*Upupa epops epops*; >81 km/day [55]), Northern Black Swifts (*Cypseloides niger borealis*; 341 km/day [56]), Red-backed Shrikes (*Lanius corullio*; 101 km/day [27]), Northern Wheatears (*Oenanthe oenanthe*; 88–160 km/day [57, 58]). The strategies used by White-crested Elaenia to cross deserts and grassland regions during fall migration was similar to that reported for species that cross the Gulf of Mexico, Mediterranean or desert regions of Africa [27, 30, 59, 60]. Our results showed that some individuals use a direct route, flying between 500–600 km/day to cross the desert and grasslands, while others take a detour, flying 100–200 km/day through areas with potentially better refueling opportunities. Birds that crossed the desert and grassland regions could be aided by tail winds. The preponderant wind in the area from where elaenias left the Patagonian Forest is known as the "Pampero", which blows in a southwest-northeast direction, with a velocity of more than 40 km/h, and which could reach southern Brazil [61, 62]. Therefore, the "Pampero" winds could serve as tail winds for birds crossing central Argentina, a pattern commonly reported for birds "en route" [63]. After crossing the desert and pampas grasslands, elaenias use different routes. Some follow the coast, crossing more grassland areas, while others begin a detour, flying through humid forests along the Paraná River, with more opportunities for refueling. For example, in the town of Capitan Bermúdez, located on the coast of Paraná River, it is common to see large numbers of White-crested Elaenias eating *Lantana* sp. fruits in March and April (Juan Carlos Teloni and Silvia María Morelli, pers. communication).

During spring migration from the Cerrado/Pantanal to the Patagonian Forest, elaenias used only the Yungas Route. This was the shortest route to the breeding areas in Patagonia. This route would also allow them to avoid head winds. Additionally, elaenias could likely refuel along this route. These three reasons may explain why all individuals used this route and could also explain why we did not find the general pattern reported in the literature, that spring migration is faster than fall migration, with birds migrating quickly in spring to arrive early at the breeding areas [29, 55,59, 64, 65].

All birds from our site in Esquel spent the winter in the same areas. They first flew to the Atlantic coast of Brazil, then to the Cerrado region of central Brazil. No birds overwintered to the west of the Andes mountain range (i.e., Peru, Ecuador and Colombia). This spatial distribution of wintering areas suggests that there is high connectivity between elaenia breeding and wintering areas, strongly linking the Patagonian forests with southern and central ecosystems of Brazil and not with Amazonia or any western ecosystem of South America, as was suggested previously (23–25). In the Cerrado region, wintering sites of individual birds were relatively close to each other. Some sites in this region could be associated with the Araguaia River and within Araguaia National Park and Araguaia State Park. In contrast, in the Atlantic Forest and Caatinga, locations were distributed along 2000 km of coast, which could be a result of habitat fragmentation. Indeed, the Atlantic Forest is the biome with the highest level of fragmentation in Brazil [66, 67].

Patagonian forests have a natural disturbance dynamic mainly driven by fire [68, 69, 70], and in recent decades the fire regime has experienced a high increase in frequency due to human activities. For example, in the last two years 5000 ha of forest were burned 30 km from our study site, in "Los Alerces" National Park, and more than 40,000 ha in 2015 in Cholila lake, 50 km from our study site. Consequently, maintaining the population size of White-crested elaenias is important for forest regeneration. For example, a reduction of 50% in elaenia abundance will not threatened this species, because of its high abundance (>8 ind/ha [18]), however, it could have a significant negative impact in forest regeneration capacity. This scenario may be plausible, because mortality rate during migration can be several times higher than during stationary periods, and fall migration may play a key role in long-term regulation of populations [71–73]. Our results indicate that this species uses the Yungas forests of northern Argentina during fall and spring migration. This biome is one of the most impacted by habitat destruction in Argentina [74]. The main cause of deforestation in Argentina throughout the last decade has been the expansion of plantations of Soybean (*Glycine max*), as well as mining and cattle production [75]. Furthermore, White-crested Elaenia overwinters in the Atlantic Forest, which is also a highly threatened biome [66, 67]. Therefore, conservation of the Atlantic Forest, Cerrado and Yungas may be critical to will not threaten population size of White-crested Elaenia, because elaenias use the three biomes sequentially.

Our results show for the first time the entire annual cycle of White-crested Elaenia; specifically, that they 1) use three different routes during fall migration, 2) that most individuals employ a counterclockwise loop migration, and 3) that they use successive winter areas probably following the fruiting of the most abundant understory species. Elaenias also exhibit high migratory connectivity between their breeding area in Patagonian Forests and wintering areas in the Atlantic Forest and Cerrado. Patagonian Forests could be affected by changes in some particular biomes of South America through links generated by White-crested Elaenia throughout their annual cycle.

## Supporting information

S1 Fig Longitude and latitude during the annual cycle. (A) Changes in longitude and (B) latitude during the annual cycle of 15 White-crested elaenias (*Elaenia albiceps chilensis*).
(JPG)

S2 Fig Heat maps: Heat maps showing the breeding site and wintering areas of 15 White-crested Elaenias. Heat map areas (ranging from black to light gray) represent 40%, 30%, 20% and 10% of data. (PDF).
(TIF)

S1 Table Departure and arrival times. Details about the fall, winter and spring migration of 15 White-crested Elaenias, including ring number (Bird ID), geolocator number (Geo ID), departure and arrival dates and route used during fall migration. Yungas Route (Y), Coast Route (C) and Paraná-Paraguay River Route (P). Since many geolocators stopped recording before arrival on the breeding site, we report the date of arrival to the Patagonian Forest biome.
^ɸ^ Birds that stopped in an area with an intermediate longitude and lower latitude than fall and winter areas (approx. 7°S 45° W) are denoted by open circles in Fig 1. The departure date from this area is noted between parentheses.
^ʄ^ Birds that spent time in the Pantanal. The departure date from this area is noted between parentheses.
(DOCX)
