# Supplementary figures and images for: Migratory timing, rate, routes and wintering areas of White-crested Elaenia (Elaenia albiceps chilensis), a key seed disperser for Patagonian forest regeneration
Source: PLoS One. 2017 Feb 9;12(2):e0170188. doi: 10.1371/journal.pone.0170188 (PMC5300243; doi:10.1371/journal.pone.0170188)

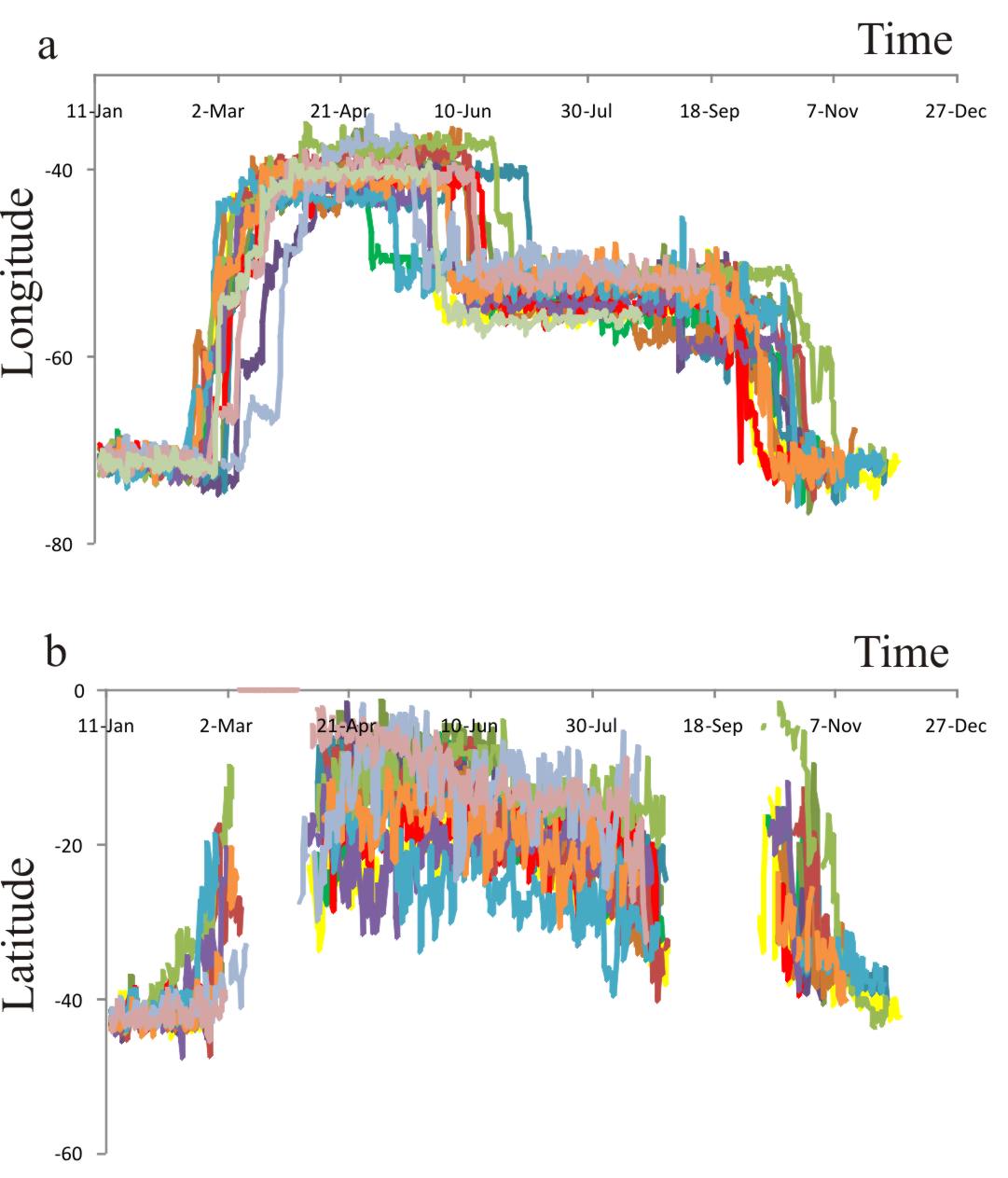

Supplement: S1 Fig — (A) Changes in longitude and (B) latitude during the annual cycle of 15 White-crested elaenias (Elaenia albiceps chilensis). (JPG) [file pone.0170188.s001.jpg]

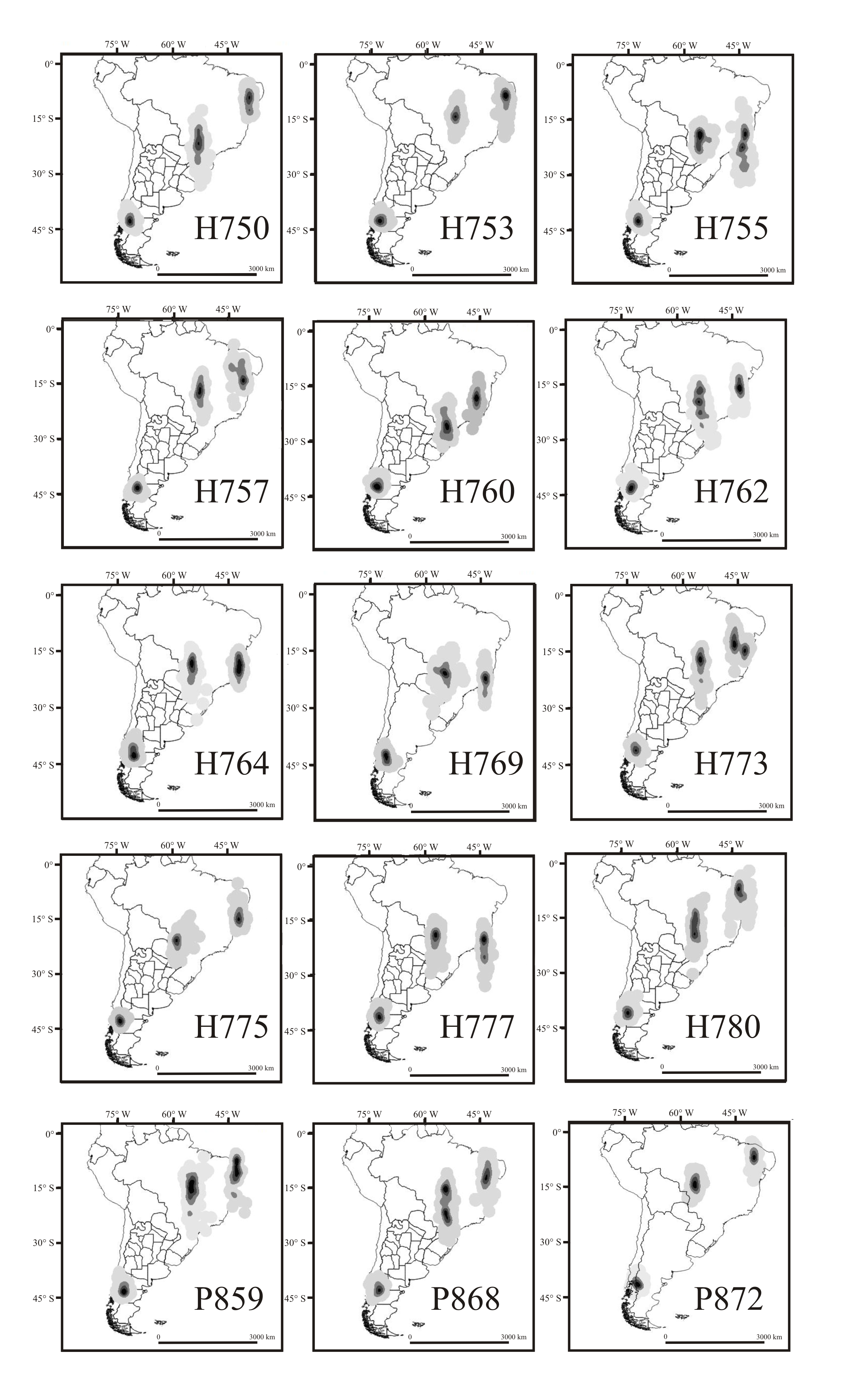

Supplement: S2 Fig — Heat map areas (ranging from black to light gray) represent 40%, 30%, 20% and 10% of data. (PDF). (TIF) [file pone.0170188.s002.TIF]
