# Supplementary material for: Migratory timing, rate, routes and wintering areas of White-crested Elaenia (Elaenia albiceps chilensis), a key seed disperser for Patagonian forest regeneration
Source: PLoS One. 2017 Feb 9;12(2):e0170188. doi: 10.1371/journal.pone.0170188 (PMC5300243; doi:10.1371/journal.pone.0170188)
Supplement: S1 Table — Details about the fall, winter and spring migration of 15 White-crested Elaenias, including ring number (Bird ID), geolocator number (Geo ID), departure and arrival dates and route used during fall migration. Yungas Route (Y), Coast Route (C) and Paraná-Paraguay River Route (P). Since many geolocators stopped recording before arrival on the breeding site, we report the date of arrival to the Patagonian Forest biome. ɸ Birds that stopped in an area with an intermediate longitude and lower latitude than fall and winter areas (approx. 7°S 45° W) are denoted by open circles in Fig 1. The departure date from this area is noted between parentheses. ʄ Birds that spent time in the Pantanal. The departure date from this area is noted between parentheses. (DOCX) [file pone.0170188.s003.docx]

**Table 1. Departure and arrival times.**

| Bird ID | Geo ID | Fall migration | | Winter migration | | Spring migration | |
| --- | --- | --- | --- | --- | --- | --- | --- |
|  |  | Departure (route) | Arrival | Departure | Arrival | Departure | Arrival Patagonian forest |
| B5589 | H753 | 21 Jan (Y) | 8 April | 22 June ^ɸ^ (27 June) | 29 June | 26 Oct | 9 Nov |
| B5597 | H760 | 11 Feb (Y) | 11 March | 12 May | 14 May | 18 Oct | 1 Nov |
| B5583 | H755 | 13 Feb (P) | 20 March | 27 May | 29 May | 5 Sept ^ʄ^ (18 Oct) | 1 Nov |
| B5595 | H764 | 17 Feb (C) | 11 April | 17 June | 19 June | 21 Sept ^ʄ^ (2 Oct) | 17 Oct |
| B5601 | H769 | 18 Feb (Y) | 3 April | 5 May ^ɸ^ (21 May) | 23 May | 1 Oct ^ʄ^ (16 Oct) | 2 Nov |
| B5578 | H762 | 20 Feb (C) | 5 April | 2 June | 4 June | 9 Oct | 22 Oct |
| B5573 | H780 | 21 Feb (C) | 10 April | 11 June ^ɸ^ (20 June) | 22 June | 13 Oct | ----- |
| B5586 | H750 | 22 Feb (P) | 11 April | 11 June | 13 June | 3 Oct | ----- |
| B5579 | H775 | 23 Feb (P) | 25 April | 14 June | 16 June | 21 Aug ^ʄ^ (5 Oct) | 24 Oct |
| B5610 | H773 | 24 Feb (C) | 8 April | 5 July | 7 July | 6 Sept ^ʄ^ (9 Oct) | 22 Oct |
| B5585 | H757 | 24 Feb (P) | 11 April | 4 June | 6 June | 28 Sept | 25 Oct |
| B5560 | P859 | 25 Feb (Y) | 17 April | 19 May | 21 May | ----- | ----- |
| B5655 | P868 | 27 Feb (Y) | 9 April | 14 June | 16 June | ----- | ----- |
| B5614 | H777 | 27 Feb (P) | 8 April | 31 May | 2 June | 5 Oct | 22 Oct |
| B5639 | P872 | 28 Feb (C) | 2 April | 29 May | 1 June | ----- | ----- |

Details about the fall, winter and spring migration of 15 White-crested Elaenias, including ring number (Bird ID), geolocator number (Geo ID), departure and arrival dates and route used during fall migration. Yungas Route (Y), Coast Route (C) and Paraná-Paraguay River Route (P). Since many geolocators stopped recording before arrival on the breeding site, we report the date of arrival to the Patagonian Forest biome.

^ɸ^ Birds that stopped in an area with an intermediate longitude and lower latitude than fall and winter areas (approx. 7°S 45° W) are denoted by open circles in Fig 1. The departure date from this area is noted between parentheses.

^ʄ^ Birds that spent time in the Pantanal. The departure date from this area is noted between parentheses.
